# A Window on the Lung: Molecular Imaging as a Tool to Dissect Pathophysiologic Mechanisms of Acute Lung Disease

**DOI:** 10.1155/2019/1510507

**Published:** 2019-08-25

**Authors:** Guido Musch

**Affiliations:** Department of Anesthesiology, Washington University in St. Louis, St. Louis, MO 63110, USA

## Abstract

In recent years, imaging has given a fundamental contribution to our understanding of the pathophysiology of acute lung diseases. Several methods have been developed based on computed tomography (CT), positron emission tomography (PET), and magnetic resonance (MR) imaging that allow regional, in vivo measurement of variables such as lung strain, alveolar size, metabolic activity of inflammatory cells, ventilation, and perfusion. Because several of these methods are noninvasive, they can be successfully translated from animal models to patients. The aim of this paper is to review the advances in knowledge that have been accrued with these imaging modalities on the pathophysiology of acute respiratory distress syndrome (ARDS), ventilator-induced lung injury (VILI), asthma and chronic obstructive pulmonary disease (COPD).

## 1. Introduction

Since the first chest X-ray, lung imaging has focused, for the most part, on detecting structural alterations of lung anatomy due to pulmonary diseases, usually inferred from abnormalities in the distribution of lung density, or changes in lung metabolism, predominantly glucose metabolism, in the diagnosis and monitoring of lung cancer with positron emission tomography (PET).

In recent years, however, there has been a tremendous growth of methods that leverage imaging to elucidate pathophysiologic mechanisms of acute lung disease. Because several of these imaging methods are noninvasive, many have been successfully translated from initial studies in animals to humans. This has allowed for a better understanding of how certain acute lung conditions develop as well as identification of which animal species, size, and injury models are most appropriate for translatability of the experimental results to the human scenario, depending on the specific condition or mechanism being investigated.

The aim of this review is to highlight a sample of acute pulmonary conditions for which imaging of experimental models has enabled substantial progress in the understanding of their pathophysiology, with a specific focus on the contributions of PET. Major relative strengths and weaknesses of the three imaging modalities discussed in this review are presented in Table 1.

## 2. Acute Respiratory Distress Syndrome (ARDS) and Ventilator-Induced Lung Injury (VILI)

### 2.1. Imaging of Pulmonary Density, Mechanics, and Inflammation

Because one of the defining features of ARDS is bilateral radiographical lung opacities, it is not surprising that imaging has played a major role in elucidating the pathophysiology of this syndrome. The current prevailing theory is that these opacities result from two main factors: (a) surfactant dysfunction [1], leading to increased surface tension at the alveolar lining and thus favoring edema and alveolar collapse and (b) the effect of gravity on the ARDS lung, with the lung's dependent portions being compressed by the weight of the overlying edema fluid [2]. Because such gravitational effect is proportional to lung height and density, animal models that reproduce this effect must have lung size and physiology similar to the human. Consequently, many studies were done in large animals such as sheep, dogs, or pigs. Although no single animal model reproduces all the complex features of ARDS pathophysiology, some established models of ARDS have been developed to reproduce its most salient features, particularly noncardiogenic pulmonary edema. These models include (1) oleic acid injection, in which oleic acid, infused through a central vein, damages the alveolocapillary membrane, thus increasing its permeability and leading to alveolar flooding [3]; (2) lung lavage, in which surfactant is depleted by repeated saline lavage, leading to unstable alveolar mechanics and development of atelectasis and edema because of increased surface tension [4]; and (3) injurious mechanical ventilation, in which high tidal volumes are used to mechanically damage the lung, leading to edema and hyaline membrane formation resembling the condition of VILI, which frequently coexists with ARDS [5–8]. Lung lavage and injurious mechanical ventilation can be combined in a “two-hit” injury model in which VILI worsens preexisting surfactant dysfunction [9].

Imaging studies performed in these animal models, mainly employing computed tomography (CT), supported the concept that, in the heterogeneously inflated ARDS lung, there is a range of opening pressures for derecruited (i.e., nonaerated) alveoli. Such pressure depends on the position of the alveolus along the gravitational axis [2], on the radius of curvature and the surface tension at the air-liquid interface, and on the energy required to fracture liquid bridges that obstruct small airways [10, 11]. By applying pressure at the airway to overcome alveolar opening pressures, either through positive end-expiratory pressure (PEEP) or recruitment maneuvers, or by changing the distribution of such pressures through prone positioning, airspaces can be recruited and lung ventilation and perfusion-to-ventilation matching restored in regions that were derecruited [12, 13]. Consequently, the first major contribution of tomographic imaging studies in animal models of ARDS is to elucidate the regional mechanism by which interventions that aim to recruit alveoli, and thus restore aeration and gas exchange, exert their beneficial effect.

CT and PET have also been used to measure the strain imposed by mechanical ventilation on different parts of the lung. Tidal lung strain is defined as the change in volume of a given region of the lung between expiration and inspiration, relative to its volume at end expiration. Strain is a key biophysical determinant of VILI. Regional tidal strain can be measured with PET imaging of inhaled [^13^N]nitrogen (^13^N_2_), by gating frame acquisition of the equilibrated tracer concentration with the end-inspiratory and end-expiratory phases. When a region of the lung expands, its gas content and hence the ^13^N_2_ concentration measured by PET increase. Thus, the change in regional tracer concentration is related to regional strain [14]. CT can measure regional tidal strain in a conceptually similar manner by measuring density changes between expiration and inspiration [15, 16] or by using registration algorithms to calculate the deformation of a given region of the lung between expiration and inspiration through a three-dimensional warping function [17].

When combined with PET imaging of 2-[^18^F]fluoro-2-deoxy-D-glucose ([^18^F]FDG) as a means to measure metabolic activation of inflammatory cells induced by mechanical ventilation [18, 19], PET/CT studies have shown that inflammation prevails in lung regions that are atelectatic or that become exposed to the largest cyclical tidal strain as the injury progresses [20, 21]. In fact, there appears to be a direct linear relationship between lung strain and [^18^F]FDG phosphorylation rate (commonly denoted as *k*_3_) [22], and [^18^F]FDG uptake rate (commonly denoted as *K*_*i*_) was increased in dependent regions of the surfactant depleted lungs, which are the regions expected to undergo repetitive collapse and reexpansion with tidal breathing [23, 24]. These findings in large animal models of VILI and ARDS have been paralleled by similar findings in patients, in whom PET/CT has revealed increased [^18^F] FDG uptake both in dense regions, where atelectasis and inflammation due to the primary etiology of ARDS are expected to predominate, and in aerated regions exposed to the iatrogenic strain of mechanical ventilation either because of tidal overdistension or cyclical alveolar recruitment and derecruitment [25–27]. Recent evidence indeed suggests that lung regions that exhibit the greatest cyclical change in density with tidal volume at the start of a period of mechanical ventilation eventually become “injured” as defined by an increase of their density above −300 Hounsfield units [28]. Importantly, regions of the lung that present increased [^18^F]FDG uptake on PET and/or CT abnormalities consistent with alterations of regional mechanical properties also reveal gene expression patterns indicative of activation of specific inflammatory pathways [20, 29]. Consequently, the second major contribution of imaging, in particular of combining the structural information derived from CT with the functional information derived from PET, is to provide mechanistic insight into the pathogenesis of VILI superimposed on ARDS and into the molecular pathways that underlie these conditions.

Recently, magnetic resonance (MR) techniques have been developed to assess geometrical and mechanical properties of the injured lungs [30, 31]. One such technique is based on the inhalation of hyperpolarized helium (^3^He). Measurement of the apparent diffusion coefficient (ADC) of ^3^He using diffusion-weighted hyperpolarized gas MR imaging yields estimates of airspace size. Using this technique, Cereda et al. [30] demonstrated that surfactant depletion by saline lavage is accompanied by an increase in alveolar size of airspaces that remain aerated, most likely as a result of distension due to parenchymal tethering to alveoli that become atelectatic. This observation suggests that alveolar derecruitment and overdistension coexist in different parts of the same lung for a given airway pressure. Application of PEEP and instillation of surfactant recruited atelectatic lung and decreased mean ADC, implying that they rendered the distribution of alveolar size more homogeneous. Another technique is based on the assessment of parenchymal elasticity by MR elastography [31].

**Figure 1:**
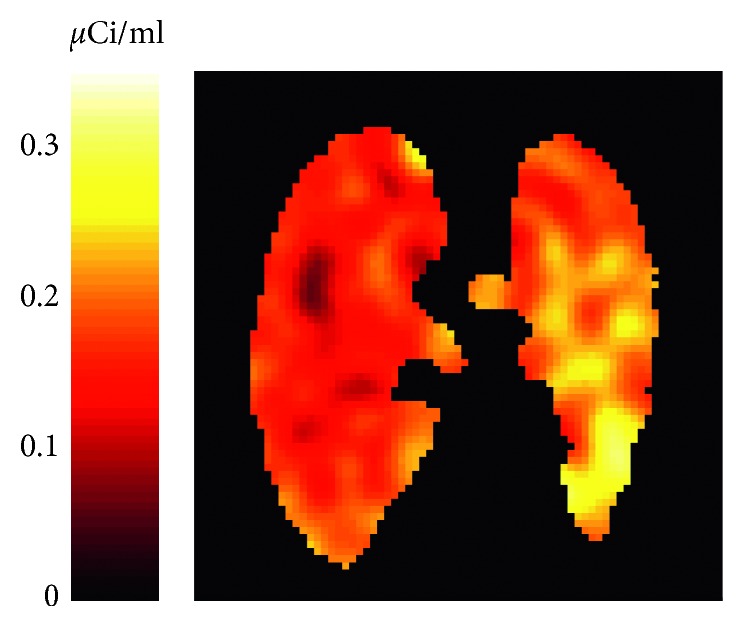
Positron emission tomography image representing pulmonary [^18^F]FDG activity 4 hours after unilateral cotton smoke inhalation to the left lung of a sheep (positioned on the right side in the figure). Note higher activity in the smoke exposed than in the control lung. Reproduced from Musch et al (Reference [34]).

PET imaging of pulmonary [^18^F]FDG uptake has also been used to detect inflammation due to sepsis and smoke inhalation, conditions that are often associated with ARDS. Studies in large animal models of sepsis using endotoxin infusion have demonstrated that inflammation in regions of high mechanical ventilatory strain is amplified by endotoxin [22, 32] and that protective mechanical ventilation with high PEEP and low tidal volume decreases [^18^F]FDG uptake in such regions [33], thus supporting the double-hit theory for the development of acute lung injury. In animal models of acute smoke inhalation, PET has been able to detect increased [^18^F]FDG uptake (Figure 1) before alterations of pulmonary gas exchange became apparent, thus lending itself to being a potentially useful early diagnostic tool for smoke inhalation-associated ARDS [34].

**Figure 2:**
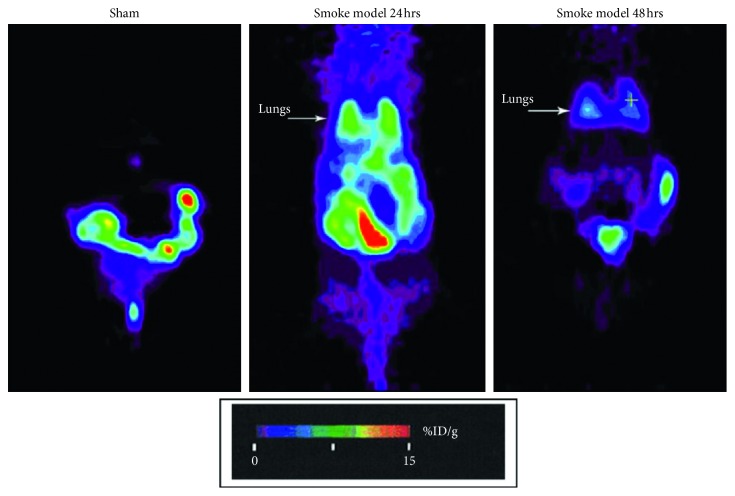
Micropositron emission tomographic image of 9-(4-[^18^F]fluoro-3-[hydroxymethyl]butyl)guanine ([^18^F]FHBG) activity in a herpes simplex virus thymidine kinase (HSV-tk) reporter mice after smoke inhalation injury. [^18^F]FHBG activity is proportional to nuclear factor-kappa B (NF-*κ*B)-mediated gene expression. The arrow indicates the location of the lungs. Note increased pulmonary NF-*κ*B activation, and hence HSV-tk expression, at 24 and 48 hours after smoke inhalation. Reproduced from Syrkina et al. (Reference [35]).

Most recently, a transgenic PET reporter mouse model of smoke inhalation has been used to demonstrate activation of nuclear factor-kappa B (NF-*κ*B) and expression of genes regulated by this transcription factor at 24 and 48 hours after acute smoke inhalation (Figure 2) [35]. Molecular imaging of endogenous gene expression requires manipulation of the animal's genome and is thus more commonly done in smaller animals, especially mice. In this technique, the herpes simplex virus thymidine kinase gene (HSV-tk) acts as the reporter transgene, inserted in the mouse genome under the control of a NF-*κ*B sensitive promoter. When NF-*κ*B is activated by an inflammatory stimulus, it translocates into the nucleus and activates transcription of all NF-*κ*B regulated genes, including the HSV-tk reporter gene. The resultant reporter protein, HSV-TK, is an enzyme that phosphorylates the acycloguanosine analog 9-(4-[^18^F]fluoro-3-[hydroxymethyl]butyl)guanine ([^18^F]FHBG). [^18^F]FHBG has two properties that make it suited for PET imaging: (1) it is a high-affinity substrate for the HSV1-TK enzyme but has relatively low affinity for mammalian thymidine kinase, resulting in improved detection sensitivity and reduced background noise [36]; (2) once phosphorylated, it is trapped inside the cell, where it accumulates proportionally to the level of HSV-tk gene expression and hence NF-*κ*B activation. This PET imaging technique thus holds promise for elucidating the sequence of molecular and genetic events that lead to the inflammatory process of ARDS and VILI noninvasively and in vivo because the same subject can be studied at multiple time points over the evolution of the acute lung condition.

### 2.2. Imaging of Pulmonary Perfusion and Gas Exchange

In addition to development of inflammation and alterations in the distribution of lung density, ARDS is characterized by alterations in the regional distribution of perfusion. Several techniques have been developed to measure regional perfusion with PET in animal models and patients with ARDS. One technique is based on the intravenous administration of ^15^O-water (H_2_^15^O) as the radiotracer [37]. Because H_2_^15^O is freely diffusible in the lung, it rapidly equilibrates between the pulmonary blood and tissue such that the concentration of tracer in the pulmonary venous blood that flows out of a region is equal to the concentration of tracer in the tissue divided by the tissue-to-blood partition coefficient of the tracer (i.e., the tracer leaves the lung at equilibrium with lung tissue). By measuring regional lung activity with PET during H_2_^15^O infusion and the subsequent equilibration phase, it is possible to calculate regional pulmonary blood flow from the equation that describes the one-compartment mathematical model of tracer distribution [37]. Regional lung water can be measured by normalizing lung tissue activity at equilibrium by the activity of blood water, measured from blood samples collected during the PET scan. Intravascular lung water can be calculated by taking a PET scan and measuring blood activity after inhalation of ^11^C- or ^15^O-carbon monoxide, which binds to hemoglobin with high affinity. Extravascular lung water can then be obtained by subtracting intravascular water from regional water. This technique thus allows determination of regional pulmonary blood flow and extravascular lung water (i.e., edema). In conditions characterized by increased pulmonary vascular permeability, such as ARDS, extravascular lung water is expected to increase [38]. A specific measure of pulmonary vascular permeability can also be derived with PET by measuring the pulmonary transcapillary escape rate of a radiolabeled protein, such as ^68^Ga-transferrin or ^11^C-methylalbumin, between the intravascular and the extravascular space [39].

Using the H_2_^15^O technique, Gust et al. [40] demonstrated that perfusion redistributes away from dependent edematous lung regions in an oleic acid-induced canine model of ARDS. This redistribution acts as a homeostatic mechanism to preserve arterial oxygenation because pulmonary perfusion is diverted away from shunting regions. Administration of intravenous endotoxin abolished this redistribution of perfusion away from dependent edematous shunting regions, thus worsening oxygenation. Because endotoxin is known to blunt hypoxic pulmonary vasoconstriction, this experimental observation implies that vascular smooth muscle contraction was responsible for the observed perfusion redistribution toward nonedematous lung regions. Interestingly, studies in patients with ARDS using the H_2_^15^O technique have also revealed lack of perfusion redistribution away from edematous regions [41], suggesting that hypoxic pulmonary vasoconstriction is, at least to some extent, impaired in ARDS, similarly to the experimental endotoxin studies. Redistribution of perfusion away from injured regions, similar to the oleic acid model, was instead demonstrated after unilateral endobronchial instillation of hydrochloric acid, a model for gastric aspiration, using PET of ^68^Ga labeled microspheres to measure regional pulmonary perfusion in rats [42].

**Figure 3:**
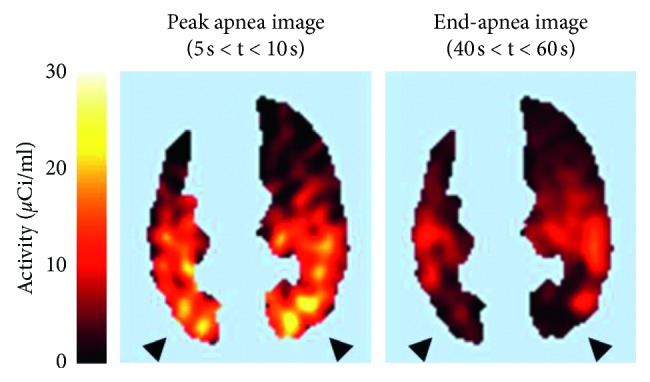
[^13^N]nitrogen (^13^N_2_) positron emission tomography images from a sheep with lavage-induced lung injury. A bolus of ^13^N_2_ in saline solution was infused intravenously over 3 seconds at the beginning of a 60-second apnea. The distribution of ^13^N_2_ during early apnea (between 5 and 10 seconds) reflects regional perfusion (peak apnea image). The distribution of ^13^N_2_ at the end of apnea (between 40 and 60 seconds) is proportional to perfusion only to aerated alveolar units, which retain ^13^N_2_ during apnea (end-apnea image). The decrease in tracer activity between peak and end-apnea images in the dorsal, dependent lung (*arrowheads*) reflects the presence of shunt in this part of the lung because alveoli that are perfused but not aerated do not retain ^13^N_2_ during apnea. Modified from Musch et al (Reference [12]).

Another PET technique to measure regional pulmonary blood flow and gas exchange is based on the intravenous administration of ^13^N_2_ in saline solution. A bolus of ^13^N_2_ gas dissolved in 20–30 ml of saline solution is infused intravenously at the beginning of a 30- to 60-second apnea while the pulmonary kinetics of ^13^N_2_ is measured by sequential PET frames. Because of the low solubility of nitrogen in blood and tissues (partition coefficient between water and air is 0.015), virtually all infused ^13^N_2_ diffuses into the alveolar airspace of aerated alveoli at first pass, where it accumulates in proportion to regional perfusion [43]. However, if alveoli are perfused but not aerated, for example, because they are atelectatic or flooded with edema, ^13^N_2_ kinetics shows an early peak of tracer activity, reflecting perfusion to that region, followed by an exponential decrease toward a plateau for the remainder of apnea. The magnitude of this decrease is proportional to regional shunt (Figure 3), and robust estimates of regional perfusion and shunt fraction can be derived by applying a mathematical model to the pulmonary kinetics of a ^13^N_2_-saline bolus, measured by PET during apnea [12]. This technique has been applied in lavage models of ARDS to elucidate the pathophysiological basis for the clinical observation that recruitment maneuvers can, at times, paradoxically worsen oxygenation by diverting perfusion toward dependent shunting lung regions [44].

## 3. Airway Obstructive Disease

Because in the ^13^N_2_-saline infusion technique the tracer is delivered to the alveolar airspace by perfusion rather than ventilation, this technique is ideally suited to quantify hypoventilation in regions of airway obstruction, which would not display a sufficient signal if delivery of tracer occurred by inhalation, as commonly done with other imaging techniques to measure regional ventilation. This characteristic makes the ^13^N_2_-saline infusion technique particularly attractive to study lung diseases characterized by bronchoconstriction.

When breathing is resumed after the end of apnea, specific alveolar ventilation (i.e., alveolar ventilation per unit of gas volume) can be calculated from the washout rate of ^13^N_2_. In the presence of uniform ventilation, the washout of tracer is accurately described by a single compartment model, manifested by single exponential washout kinetics [43]. In contrast, in the presence of intraregional heterogeneity of ventilation, as is usually found in airway obstructive diseases such as asthma and chronic obstructive pulmonary disease (COPD), the washout kinetics is better described by a multicompartmental model, with a fast and a slow ventilating compartments that represent alveolar units with, respectively, normal ventilation and hypoventilation.

Using this multicompartmental model, Vidal Melo et al. [45] were able to derive ventilation-perfusion distributions from PET images of ^13^N_2_ that allowed accurate estimation of blood gases in animal models of asthma by inhaled methacholine. The bimodality of these distributions during bronchoconstriction reflected hypoventilation of large contiguous regions of the lung [46], a finding that was confirmed in patients with asthma [47]. In these states, the fraction of lung volume presenting intraregional ventilation heterogeneity (i.e., multicompartmental ^13^N_2_ washout kinetics) is substantial, in contrast to the predominant single compartment behavior of normal lungs [43].

The extreme case of hypoventilation is represented by airway closure leading to gas trapping distal to the occluded airway. Gas trapping regions will appear as regions of ^13^N_2_ retention on PET frames acquired at the end of the washout phase. This characteristic has been leveraged to demonstrate that the prone position is effective in reducing areas of gas trapping in asthmatic subjects with induced bronchoconstriction [48].

Combined measurements of regional perfusion and ventilation in patients with COPD showed that both ventilation and perfusion are more heterogeneously distributed in COPD than in normal subjects. However, the heterogeneity of perfusion was greater than expected from the increase in ventilation heterogeneity and occurred predominantly at large length scales, suggesting that perfusion heterogeneity could serve as an early biomarker of pulmonary vascular involvement in COPD [49].

## 4. Conclusion

In recent years, pulmonary structural and functional imaging techniques based on CT, PET, and MR have been applied to animal models of ARDS (oleic acid infusion and saline lung lavage), sepsis (endotoxin infusion), VILI (high tidal volume mechanical ventilation), and asthma (methacholine inhalation), yielding fundamental progress in our understanding of the pathophysiology of these conditions. Because of their noninvasive nature, several of these techniques can be translated to the corresponding human condition, thus enhancing the clinical relevance of these animal studies.

## Figures and Tables

**Table 1:** Main strengths and weakness of CT, PET, and MR for functional lung imaging.

	Strengths	Weaknesses
CT	High spatial resolution	Radiation exposure
	Speed of acquisition	Limited ability to image biologic processes

PET	Image biologic processes	Radiation exposure
	Tracer kinetic modeling	Lower spatial resolution

MR	High spatial resolution	Requires hyperpolarized gases to image ventilation
	Radiation free	Limited ability to image biologic processes
